# Supplementary material for: Predicting chemoresponsiveness in epithelial ovarian cancer patients using circulating small extracellular vesicle-derived plasma gelsolin
Source: J Ovarian Res. 2023 Jan 16;16:14. doi: 10.1186/s13048-022-01086-x (PMC9841140; doi:10.1186/s13048-022-01086-x)
Supplement: Supplementary file 1 — Additional file 1: Supplementary Figure 1. Distribution of individual biomarkers between chemoresistant (PFI ≤ 6 months) and chemosensitive (PFI > 6 months) groups using dot plots. Points on dot plots represent individual patient biomarker concentrations. Line with error bars represent group mean and SEM. (A) CA125, Mann-Whitney U-test. (B) Total pGSN, Student t-test. (C) sEV-pGSN, Mann-Whitney U-test. Supplementary Figure 2. Kaplan-Meier survival analysis (log-rank test) between total pGSN, sEV-pGSN, or total pGSN/sEV-pGSN with DFS or OS. Cut-off values were determined using Fisher’s exact test. (A) Total pGSN, DFS. (B) Total pGSN, OS. (C) sEV-pGSN, DFS. (D) sEV-pGSN, OS. (E) Total pGSN/sEV-pGSN, DFS. (F) Total pGSN/sEV-pGSN, OS. Supplementary Figure 3. Distribution of multi-analyte biomarkers between chemoresistant (PFI ≤ 6 months) and chemosensitive (PFI > 6 months) groups using dot plots. Points on dot plots represent individual patient biomarker concentrations. Line with error bars represent group mean and SEM. (A) Total pGSN/sEV-pGSN. (B) Total pGSN/CA125. (C) sEV-pGSN/CA125. (D) (Total pGSN/sEV-pGSN)/CA125. Mann-Whitney U-test used for all four multi-analyte biomarkers. Supplementary Figure 4. Receiver operating characteristic curve analysis for individual and multi-analyte biomarkers to predict PFI ≤ 6 months. (A) Total pGSN, sEV-pGSN, and total pGSN/sEV-pGSN. (B) CA125, total pGSN/CA125, sEV-pGSN/CA125, and (total pGSN/sEV-pGSN)/CA125. Supplementary Figure 5. Characterization of sEVs. (A) Western blot of sEV surface markers (CD9, CD63, and CD81), a cytoplasmic marker (GAPDH), and a negative sEV marker (calnexin). (B) Electron micrograph illustrating pGSN within extracellular vesicles. (C) Size distribution curve from nanoparticle tracking analysis of 5 representative samples. Bars represent mean particle count and error bars represent SEM. [file 13048_2022_1086_MOESM1_ESM.docx]

**Supplementary Figures:**


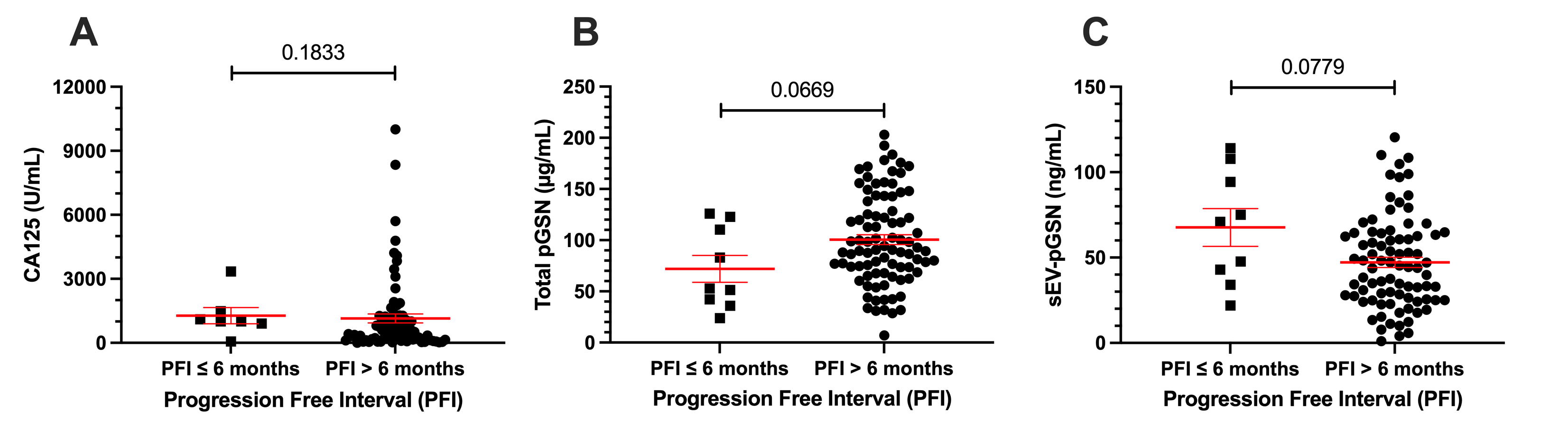


**Supplementary Figure 1.** Distribution of individual biomarkers between chemoresistant (PFI ≤ 6 months) and chemosensitive (PFI > 6 months) groups using dot plots. Points on dot plots represent individual patient biomarker concentrations. Line with error bars represent group mean and SEM. **(A)** CA125, Mann-Whitney U-test. **(B)** Total pGSN, Student t-test. **(C)** sEV-pGSN, Mann-Whitney U-test.


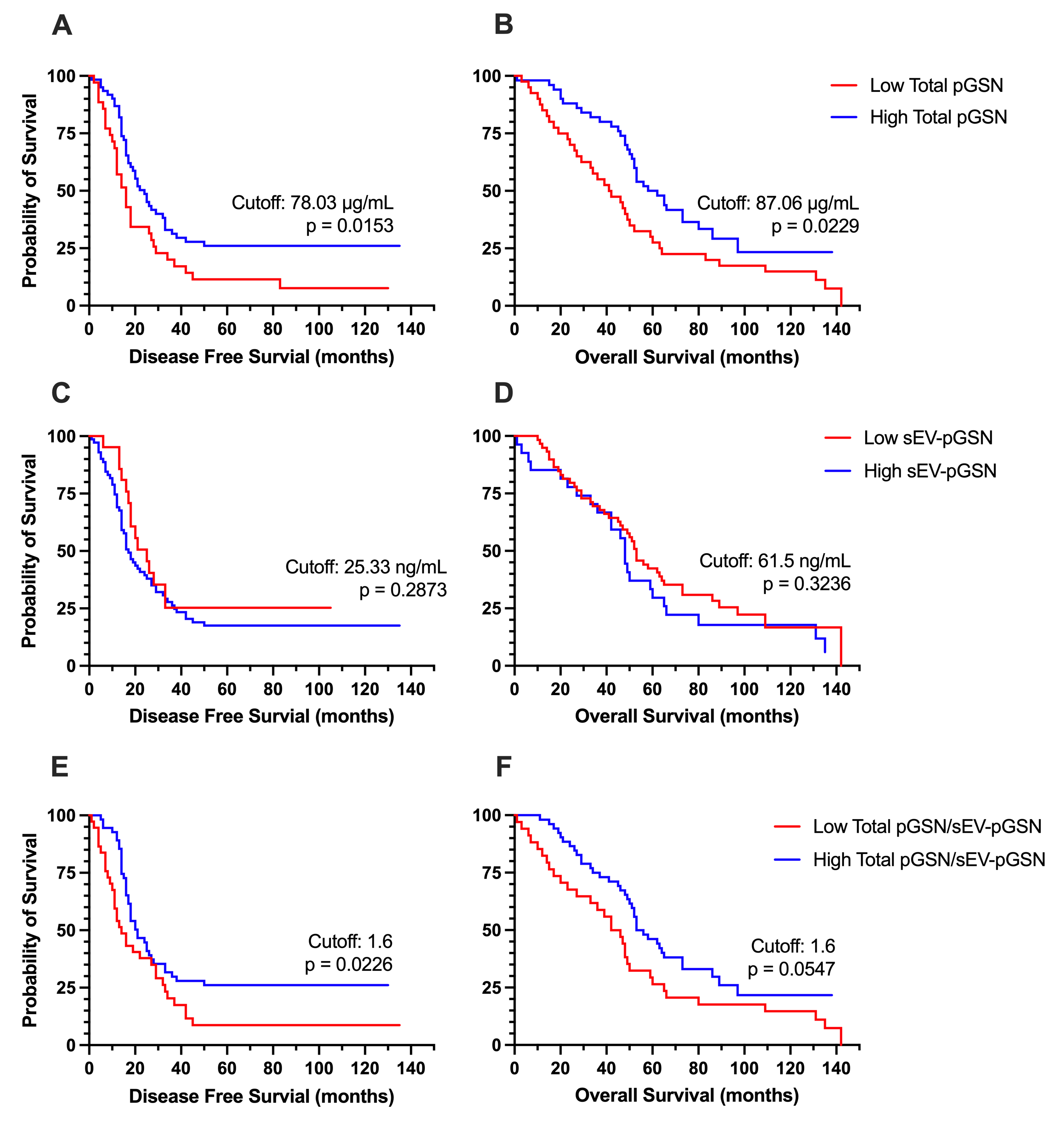


**Supplementary Figure 2.** Kaplan-Meier survival analysis (log-rank test) between total pGSN, sEV-pGSN, or total pGSN/sEV-pGSN with DFS or OS. Cut-off values were determined using Fisher’s exact test. **(A)** Total pGSN, DFS. **(B)** Total pGSN, OS. **(C)** sEV-pGSN, DFS. **(D)** sEV-pGSN, OS. **(E)** Total pGSN/sEV-pGSN, DFS. **(F)** Total pGSN/sEV-pGSN, OS.


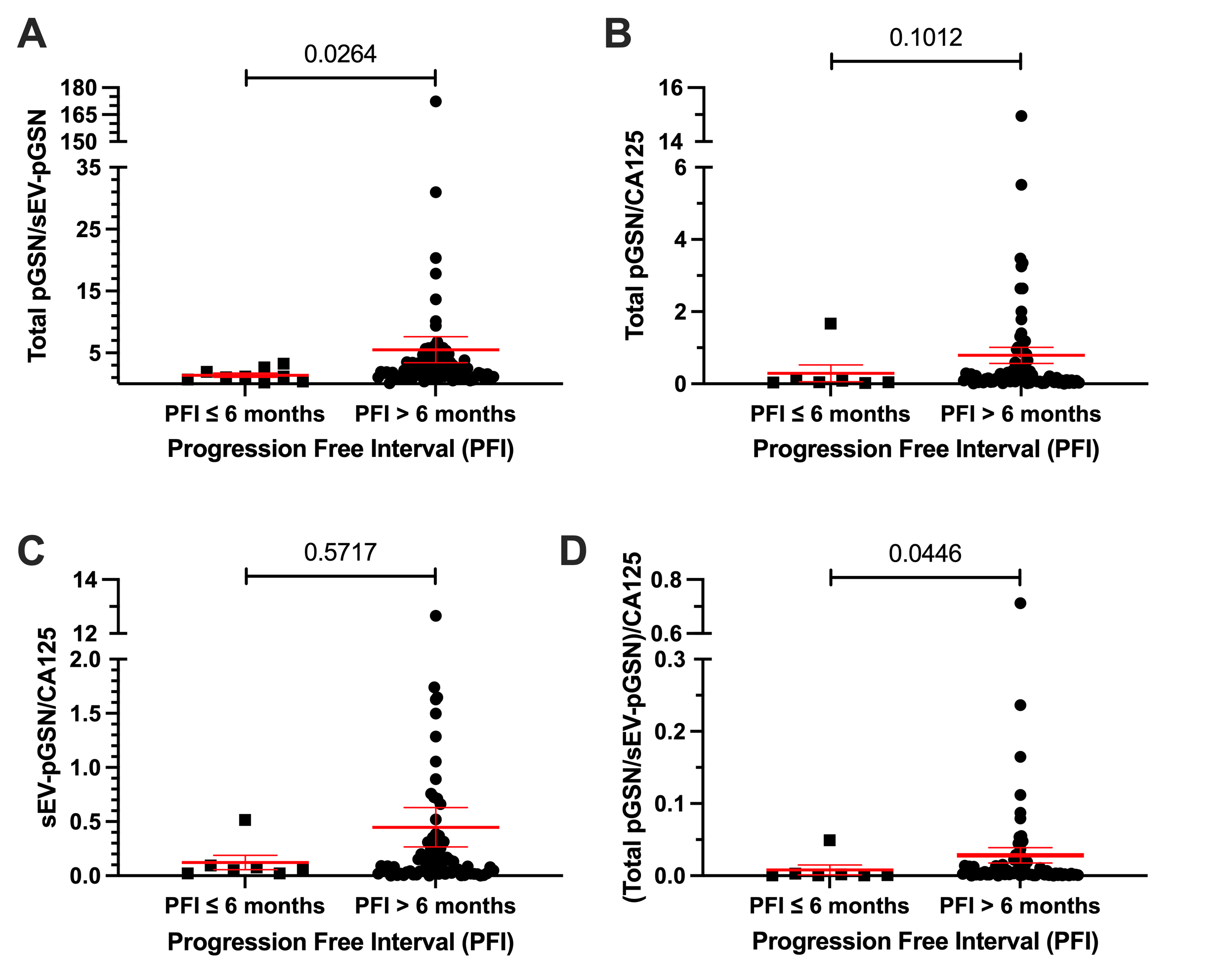


**Supplementary Figure 3.** Distribution of multi-analyte biomarkers between chemoresistant (PFI ≤ 6 months) and chemosensitive (PFI > 6 months) groups using dot plots. Points on dot plots represent individual patient biomarker concentrations. Line with error bars represent group mean and SEM. **(A)** Total pGSN/sEV-pGSN. **(B)** Total pGSN/CA125. **(C)** sEV-pGSN/CA125. **(D)** (Total pGSN/sEV-pGSN)/CA125. Mann-Whitney U-test used for all four multi-analyte biomarkers.


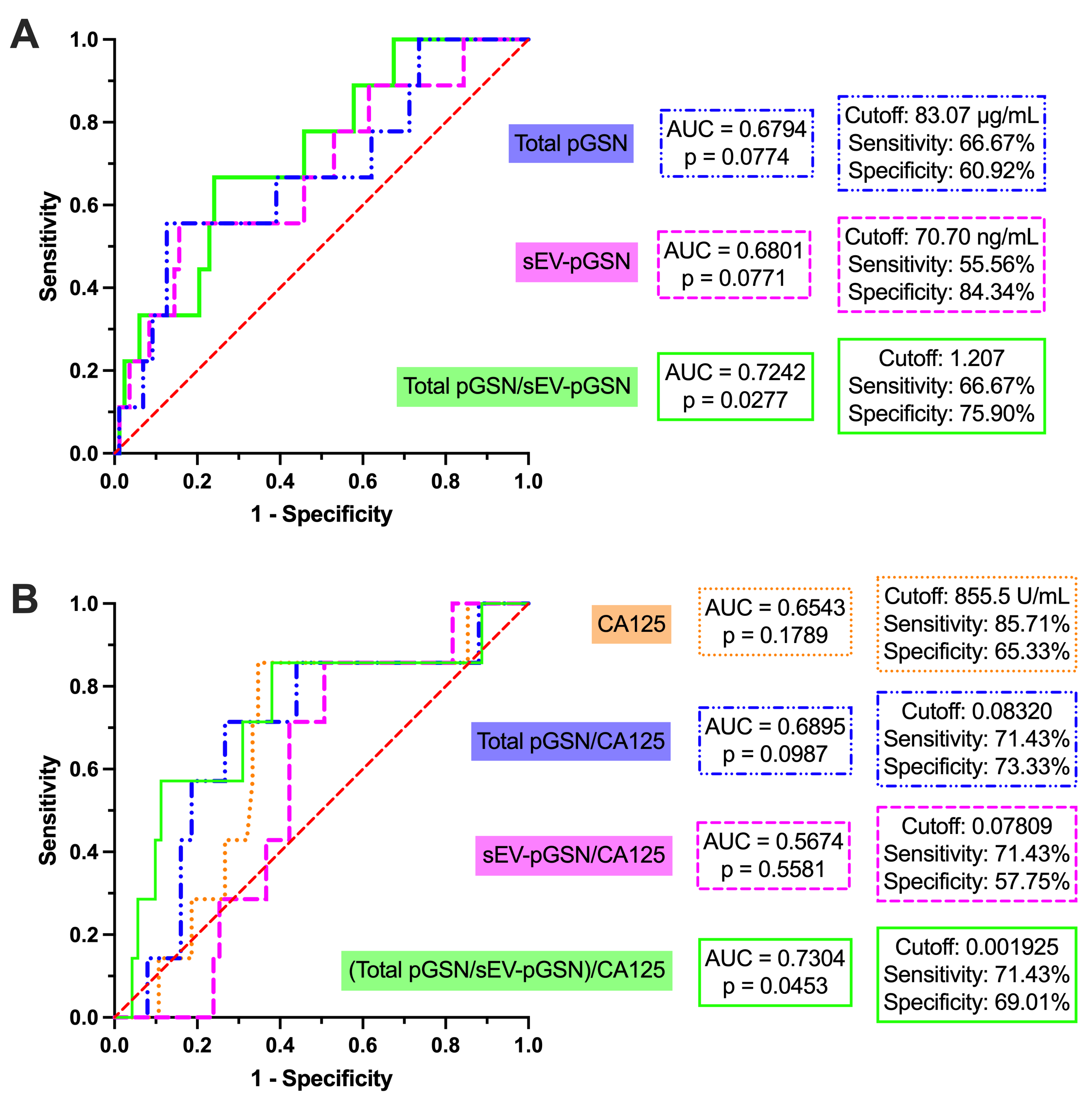


**Supplementary Figure 4.** Receiver operating characteristic curve analysis for individual and multi-analyte biomarkers to predict PFI ≤ 6 months. **(A)** Total pGSN, sEV-pGSN, and total pGSN/sEV-pGSN. **(B)** CA125, total pGSN/CA125, sEV-pGSN/CA125, and (total pGSN/sEV-pGSN)/CA125.


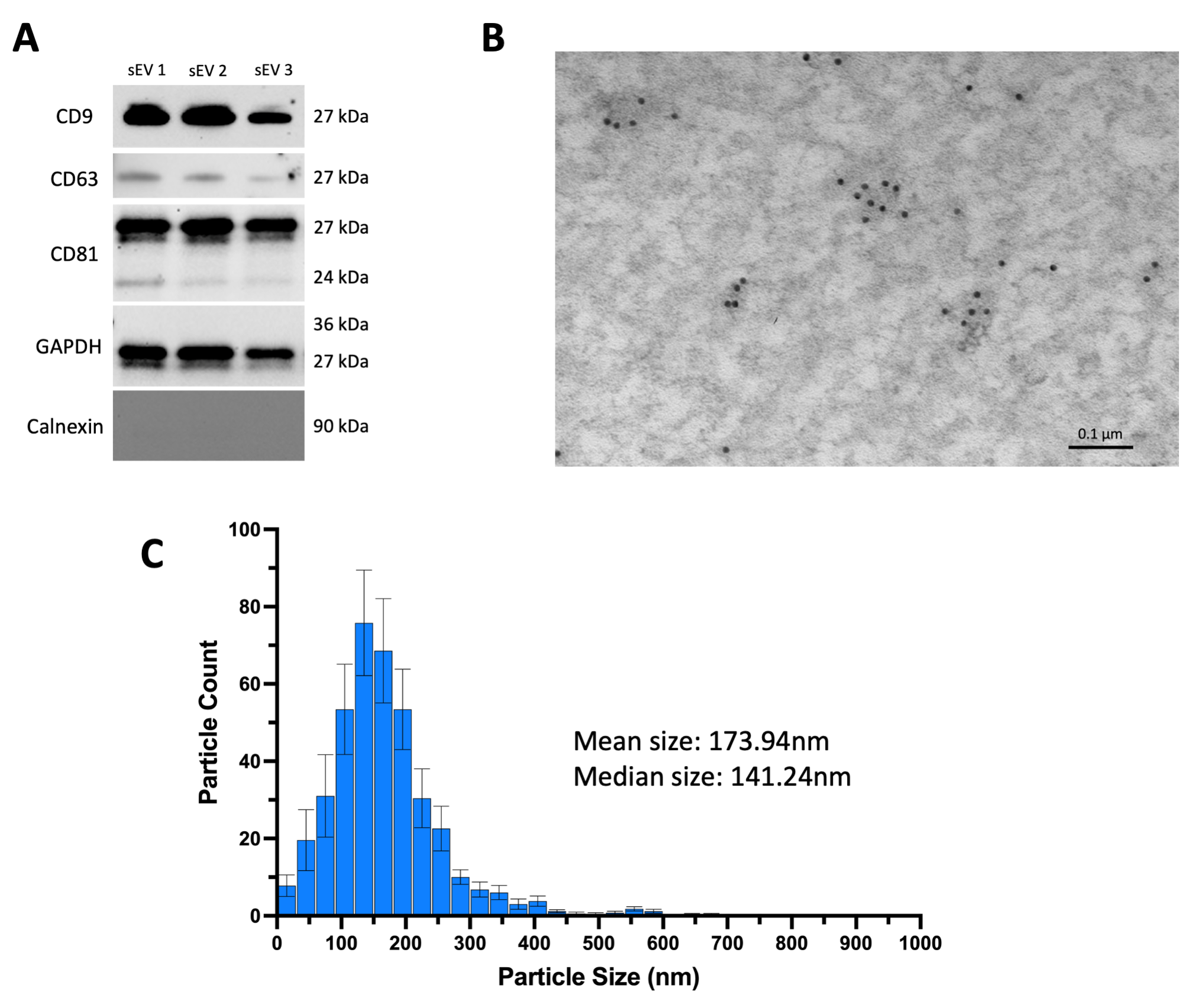


**Supplementary Figure 5.** Characterization of sEVs. (A) Western blot of sEV surface markers (CD9, CD63, and CD81), a cytoplasmic marker (GAPDH), and a negative sEV marker (calnexin). (B) Electron micrograph illustrating pGSN within extracellular vesicles. (C) Size distribution curve from nanoparticle tracking analysis of 5 representative samples. Bars represent mean particle count and error bars represent SEM.
